# Data on DOC and N from the Muz taw glacier in Central Asia

**DOI:** 10.1016/j.dib.2020.105556

**Published:** 2020-04-18

**Authors:** Tanguang Gao, Shichang Kang, Yulan Zhang, Michael Sprenger, Feiteng Wang, Wentao Du, Xiaoming Wang, Xiaoxiang Wang

**Affiliations:** a Key Laboratory of Western China's Environmental Systems (Ministry of Education), College of Earth and Environmental Sciences, Lanzhou University, Lanzhou 730000, China; b State Key Laboratory of Cryospheric Science, Northwest Institute of Eco-Environment and Resources, Chinese Academy of Sciences, Lanzhou 730000, China; c CAS Centre for Excellence in Tibetan Plateau Earth Sciences, Chinese Academy of Sciences, Beijing 100101, China; d Institute for Atmospheric and Climate Science, ETH Zurich, CH-8092 Zurich, Switzerland

**Keywords:** Dissolved organic carbon, Total dissolved nitrogen, Glacier melt, Radiative forcing, Central asia

## Abstract

This Data in Brief article provides a supplementary information to the dissolved organic carbon and nitrogen from the snow of Muz taw glacier in the Central Asia, which is related to the scientific article titled with “Characterization, sources and transport of dissolved organic carbon and nitrogen from a glacier in the Central Asia”[1]. Meanwhile, major ions (including Na^+^, *K*^+^, NH_4_^+^, Ca^2+^, Mg^2+^, Cl^−^, SO_4_^2−^, NO_3_^−^, and NO_2_^−^) were also reported. These data were analysed using descriptive statistics such as correlations and principle component analysis. Additionally, we conducted a literature review on DOC and N concentrations for the comparison. This article also presents the analysis data of the mass absorption cross section of DOC in snow.

Specifications table

**Table:** 

Subject	Earth and Planetary Sciences
Specific subject area	Geophysics, Atmospheric Science
Type of data	Table
How data were acquired	Measurements, Microsoft Excel 2013
Data format	Raw, Analysed, Graphs
Parameters for data collection	Data were obtained by analysis using equipment in the clean laboratory room. Pre-cleaned polycarbonate bottles were used to keep the snow samples according to the standard “Clean Hands-Dirty Hands” protocol.
Description of data collection	DOC and TDN were analysed by using a total organic carbon analyser. Major ions were analysed by using ion chromatography.
Data source location	Lanzhou University, Lanzhou, China
	Muz Taw glacier in Sawir Mountains of Central Asia (No. 5A259C0001, 47.06 °N, 85.56 °E)
Data accessibility	With the article
Related research article	Gao, T., Kang, S., Zhang, Y., Sprenger, M., Wang, F., Du, W.., Wang, X.X., Wang X.M. Characterization, sources and transport of dissolved organic carbon and nitrogen from a glacier in the Central Asia. Science of the Total Environment, STOTEN_138,346 (accepted).

## 1 Value of the data

The obtained data expand the understanding of DOC and N in glaciers of Central Asia.
These data can be of benefit to other researchers.
These data can be used for estimation of DOC and N export from glacier melting.
The data provide a basis to further insight on carbon and nitrogen cycles in the cryopsheric region.

## 1 Data description

**Fig. 1:**
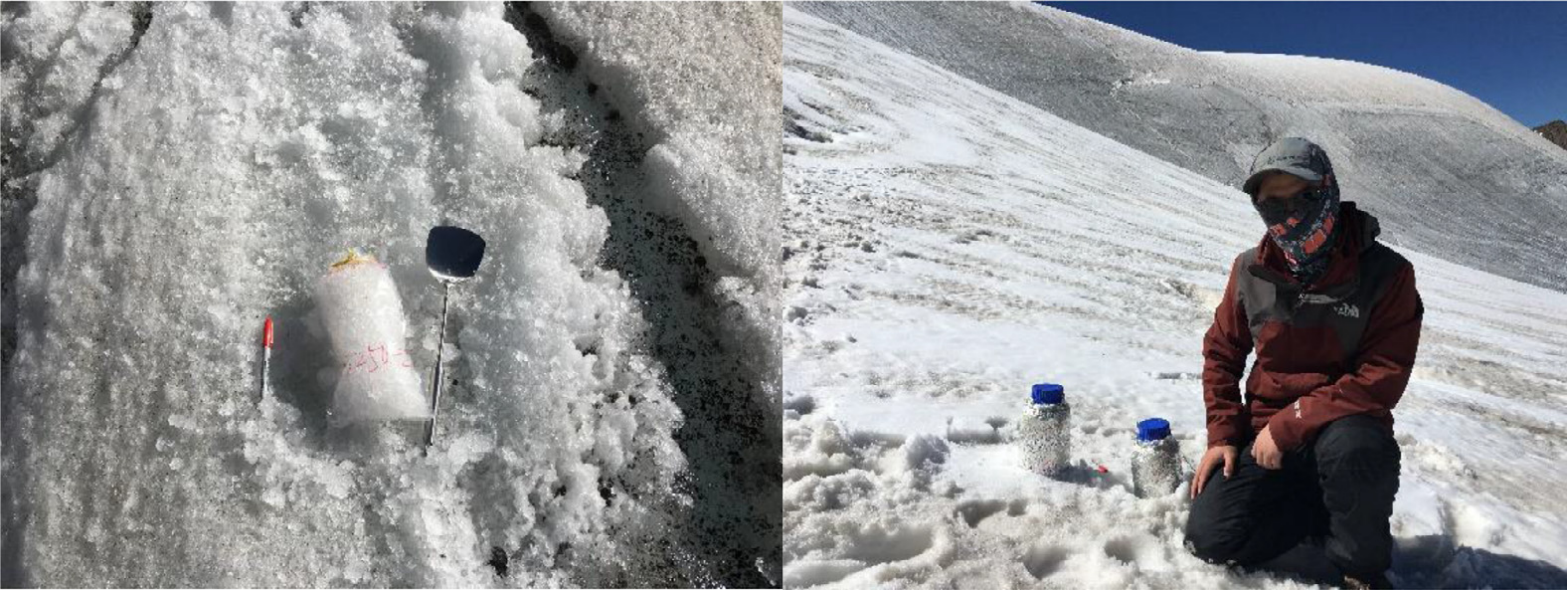
Field work in the Muz Taw glacier.

**Fig. 2:**
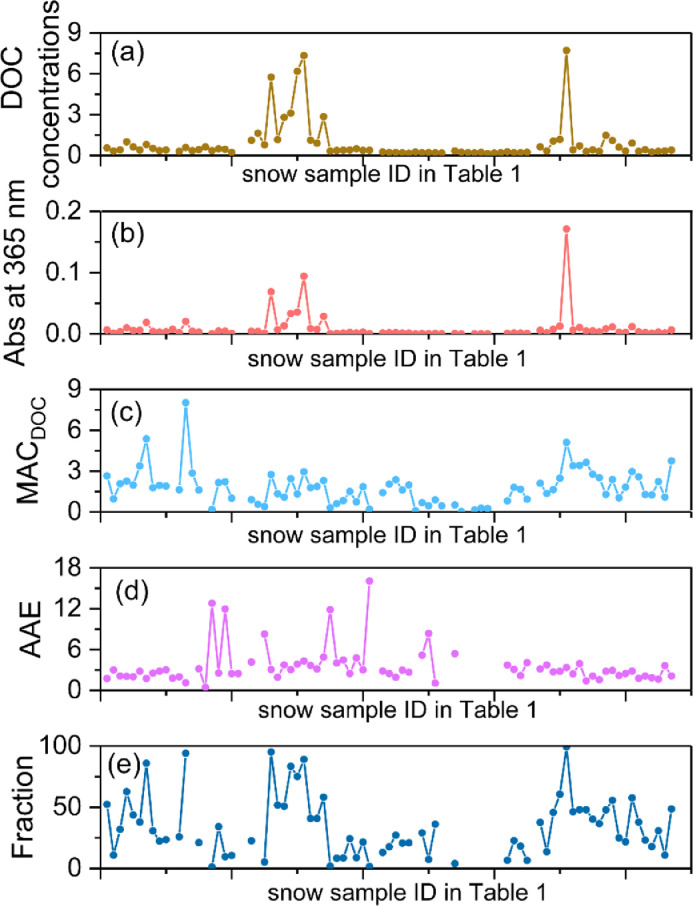
Data of DOC, and the calculated AAE, MAC, and fraction of RF by DOC relative to RF by BC in snow of Muz taw glacier.

The data set of this article contained information on the characteristics of DOC, TDN, DIN, DON, and major ions in surface aged snow and snowpit from Muz Taw glacier collected in 2018 in central Asia (Table 1). Fig. 1 shows the field work on the snow samples collection from the Muz Taw glacier. The Fig. 2 shows the experimental data on the light absorption of DOC in snow at the wavelength of 365 nm (abs at 365) and the calculated AAE, MAC, and fraction of radiative forcing (RF) by DOC relative to RF by BC in snow.

**Table 1:** The raw data of DOC, TDN, DIN,DON, and major ions in snow collected from Muz Taw glacier in 2018, in Central Asia. (glacier No. 5A259C0001, Lat. 47.06 °N, Lon. 85.56 °E).

Sample ID	Na+ (ppm)	NH4+ (ppm)	*K*+ (ppm)	Ca2+ (ppm)	Mg2+ (ppm)	CL- (ppm)	SO42- (ppm)	NO3- (ppm)	NO2- (ppm)	DOC (ppm)	TDN (ppm)	DIN (ppm)	DON (ppm)
MSD1-1	0.2100	0.2851	0.0539	0.3680	0.0927	0.0778	0.0703	0.0436	0.0122	0.5568	0.4628	0.2353	0.2275
MSD1-2	0.1697	0.3152	0.0461	0.3320	0.0598	0.0923	0.1533	0.0435	0.0119	0.3207	0.3876	0.2586	0.1290
MSD1-3	0.2162	0.5300	0.0669	0.3372	0.0623	0.1218	0.1243	0.0794	0.0161	0.4069	0.6238	0.4350	0.1888
MSD1-4	0.3968	0.8148	0.1362	0.5717	0.1353	0.2720	0.2223	0.0529	0.0122	1.0040	0.9963	0.6494	0.3469
MSD1-5	0.3206	0.4133	0.0610	0.5068	0.1062	0.1974	0.1100	0.0534	0.0080	0.6281	0.5965	0.3359	0.2606
MSD1-6	1.2175	0.4347	0.1020	0.4493	0.0959	0.0376	0.1565	0.0468	0.0161	0.3977	0.5170	0.3536	0.1634
MSD1-7	0.7610	0.5182	0.0720	0.3595	0.0924	0.0435	0.1352	0.0611	0.0169	0.8036	0.7083	0.4220	0.2863
MSD1-8	0.1645	0.4350	0.0465	0.3342	0.0779	0.0947	0.1623	0.0448	0.0131	0.5175	0.5403	0.3524	0.1879
MSD1-9	0.1430	0.2007	0.0415	0.2334	0.0489	0.0375	0.0439	0.0257		0.3471	0.2657	0.1619	0.1038
MSD1-10	0.1396	0.4129	0.0476	0.4233	0.0639	0.0523	0.0637	0.0460	0.0133	0.4003	0.4584	0.3356	0.1228
MSD1-11	0.1489	0.2920	0.0243	0.3868	0.0530	0.0542	0.2590	0.0556	0.0121	0.2971	0.3797	0.2433	0.1364
MSD1-12	0.2187	0.5472	0.0636	0.5040	0.1099	0.0991	0.1778	0.0516	0.0114	0.5853	0.6302	0.4407	0.1895
MSD1-13	0.1126	0.2706	0.0254	0.2593	0.0446	0.0469	0.0850	0.0345	0.0194	0.3491	0.4004	0.2242	0.1762
MSD1-14	0.1962	0.4071	0.0343	0.4106	0.0768	0.0964	0.2874	0.0606	0.0118	0.4286	0.5220	0.3339	0.1881
MSD1-15	0.2258	0.5002	0.0662	0.4533	0.1076	0.1139	0.0827	0.0468		0.6326	0.5919	0.3996	0.1923
MSD1-16	0.1323	0.1820	0.0208	0.2448	0.0431	0.0346	0.0511	0.0354	0.0139	0.3524	0.2967	0.1538	0.1429
MSD1-17	0.1247	0.2284	0.0233	0.2638	0.0465	0.0139	0.0729	0.0260	0.0214	0.4918	0.5126	0.1900	0.3226
MSD1-18	1.8783		0.0569	0.4634	0.0955	0.0317	0.0836	0.0456	0.0207	0.4532	0.4828	0.0166	0.4662
MSD1-19	0.0745	0.1302	0.0116	0.1185	0.0154	0.0298	0.0675	0.0256	0.0076	0.2190	0.2943	0.1094	0.1849
MSD3116-1	1.9474	1.5048	0.4945	1.3932	0.1141	0.0739	0.4130	0.0272	0.0118	1.1080	1.5000	1.1801	0.3199
MSD3116-2	1.9121	0.2954	0.2834	0.6442	0.0818	0.2427	0.5400		0.0150	1.6330	0.4692	0.2343	0.2349
MSD3216-1	1.7701	1.3935	1.0780	0.5741	0.1186	0.1110	0.3193	0.0145	0.0118	0.7809	0.8007	1.0907	
MSD3216-2	0.9843	0.6428	0.2680	0.8852	0.1135	0.1122	0.1872	0.0658	0.0128	5.7500	1.7620	0.5187	1.2433
MSD3266-1	2.8049	1.1914	0.8060	2.4018	0.3186	0.0446	0.1898	0.0145		1.1590	1.6910	0.9299	0.7611
MSD3266-2	2.0856	0.6972	0.7819	3.7463	0.1148	0.0657	0.2489	0.0145		2.8000	1.4660	0.5456	0.9204
MSD3266-3	1.8163	0.9134	0.4461	0.4998	0.0972	0.0326	0.1777	0.0125		3.1070	1.0050	0.7133	0.2917
MSD3316-1	3.1106	2.0719	1.7264	5.2593	0.2625	0.0780	0.2849	0.0499	0.0159	6.1750	3.5440	1.6276	1.9164
MSD3316-2	4.6492	0.5942	0.8144	1.2551	0.1435	0.0322	0.1789	0.0132		7.3370	1.0940	0.4651	0.6289
MSD3316-3	1.1542	0.1589	0.1057	0.4024	0.0619	0.0341	0.0827	0.0243		1.1170	0.3312	0.1291	0.2021
MSD3316-4	1.9305	0.4410	0.1318	0.6422	0.1073	0.0213	0.0728	0.0286	0.0117	0.9003	0.4824	0.3530	0.1294
MSD3366-2	1.7580	0.7848	0.6977	0.5316	0.1203	0.0651	0.2024	0.0132		2.8480	1.1333	0.6134	0.5199
MSD3366-3	0.6376		0.0236	2.8782	0.0336	0.0136	0.0487	0.0223	0.0191	0.3210	0.1613	0.0109	0.1504
MSD3416-1	0.4308	0.1964	0.0656	0.2255	0.0488	0.0110	0.1126	0.0511	0.0171	0.3644	0.3139	0.1695	0.1444
MSD3416-2	0.4304	0.0388	0.0295	1.3250	0.0342	0.0125	0.0666	0.0216		0.3843	0.3526	0.0350	0.3176
MSD3416-3	0.6926		0.0208	1.2682	0.0353	0.0154	0.1037	0.0435	0.0147	0.3984	0.3280	0.0143	0.3137
MSD3466	1.0747	0.2411	0.0596	0.4549	0.0831	0.0175	0.1192	0.0400		0.4960	0.3130	0.1966	0.1164
MSD3466-2	1.1547	0.2496	0.0527	0.4798	0.1475	0.0295	0.1034	0.0472	0.0124	0.3692	0.3223	0.2086	0.1137
MSD3516	0.6759	0.1110	0.0324	0.2550	0.0548	0.0113	0.1318	0.0527	0.0149	0.3799	0.2973	0.1028	0.1945
MSDSP0-10	0.8173		0.0245	0.3022	0.0530	0.0728	0.0558	0.0246	0.0133	0.2551	0.0997	0.0096	0.0901
MSDSP10-20	0.6476	0.1233	0.0216	0.2652	0.0555	0.0898	0.1505	0.0720		0.2175	0.2624	0.1122	0.1502
MSDSP20-30	0.7258	0.0669	0.0230	0.2260	0.0442	0.0309	0.0982	0.0419	0.0119	0.2159	0.1654	0.0651	0.1003
MSDSP30-40	0.7972	0.1519	0.0644	0.3285	0.0481	0.0566	0.1796	0.0667	0.0119	0.1984	0.3077	0.1368	0.1709
MSDSP40-50	0.7655	0.1469	0.0326	0.2550	0.0383	0.0540	0.1138	0.0578	0.0272	0.1626	0.2802	0.1356	0.1446
MSDSP50-60	0.6998	0.1609	0.0205	0.2992	0.0417	0.0345	0.1741	0.0597		0.2507	0.2133	0.1387	0.0746
MSDSP60-70	0.6956	0.1253	0.0105	0.2439	0.0315	0.0263	0.0629	0.0438		0.2030	0.1243	0.1073	0.0170
MSDSP70-80	0.5904	0.2393	0.0143	0.2793	0.0290	0.0425	0.1471	0.0822		0.2180	0.2857	0.2047	0.0810
MSDSP80-90	0.4314	0.1547	0.0109	0.2018	0.0299	0.0678	0.1102	0.0439	0.0075	0.1998	0.3716	0.1325	0.2391
MSDSP90-100	0.4271	0.2213	0.0149	0.2391	0.0216	0.0330	0.1973	0.0815		0.1787	0.4455	0.1905	0.2550
MSD3100-1	0.6205		0.0749	2.2790	0.0388	0.0406	0.1171	0.0419	0.0151	0.6315	0.3067	0.0141	0.2926
MSD3100-2	0.5787	0.2407	0.0326	0.2423	0.0302	0.0095	0.1387	0.0589	0.0150	0.3305	0.3083	0.2051	0.1032
MSD3150-1	1.2828	0.4864	0.1245	0.3920	0.0628	0.0341	0.1101	0.0725		1.0560	0.5717	0.3947	0.1770
MSD3150-2	2.9400	0.2640	0.5594	2.3884	0.3758	1.0140	0.6595	0.1175	0.0211	1.1790	0.5165	0.2383	0.2782
MSD3200	2.3019	0.8469	0.9252	4.4092	0.1483	0.0575	0.3699	0.0196	0.0159	7.7090	1.9330	0.6680	1.2650
MSD3200-1	0.6475		0.0485	3.3634	0.0349	0.0119	0.0867	0.0222		0.4108	0.2348	0.0050	0.2298
MSD3200-2	0.4473	0.0959	0.0405	0.2155	0.0306	0.0358	0.0479	0.0266	0.0112	0.7009	0.2576	0.0840	0.1736
MSD3250-1	0.6912	0.0917	0.0554	0.6841	0.0693	0.0401	0.1567	0.0335	0.0141	0.2915	0.1373	0.0832	0.0541
MSD3250-2	0.8698	0.2974	0.0445	0.6031	0.0569	0.0274	0.0626	0.0476	0.0137	0.4056	0.3436	0.2462	0.0974
MSD3300-1	0.5331	0.1138	0.0356	0.2638	0.0378	0.0029	0.1061	0.0314	0.0118	0.2989	0.2029	0.0992	0.1037
MSD3300-2	1.2754	1.7762	0.9672	1.7392	0.1705	0.0294	0.1985	0.0154		1.4780	1.8500	1.3849	0.4651
MSD3350-1	1.4671		0.1703	3.9663	0.0768	0.0111	0.1051	0.0177	0.0134	1.0950	0.3631	0.0081	0.3550
MSD3350-2	0.7458	0.0050	0.0475	5.6400	0.0585	0.0141	0.0583	0.0205	0.0119	0.6084	0.2755	0.0121	0.2634
MSD3400-1	0.6787	0.1571	0.0228	0.3821	0.0443	0.0209	0.0493	0.0221	0.0162	0.3232	0.1938	0.1321	0.0617
MSD3400-2	0.7126	0.0931	0.1511	0.3920	0.0582	0.0515	0.0895	0.0314	0.0179	0.9053	0.8295	0.0849	0.7446
MSD3450-1	0.9045	0.1156	0.0133	0.2910	0.0383	0.0102	0.1450	0.0355		0.3039	0.1742	0.0979	0.0763
MSD3450-2	0.7442	0.3894	0.0843	0.4958	0.0563	0.0118	0.1098	0.0312	0.0134	0.4224	0.4282	0.3140	0.1142
MSD3500-1	0.7859	0.1840	0.0655	0.3661	0.0593	0.0143	0.0679	0.0462	0.0160	0.2480	0.2619	0.1584	0.1035
MSD3500-2	0.7449	0.1258	0.0128	0.2578	0.0360	0.0064	0.0801	0.0366		0.3032	0.1277	0.1061	0.0216
MSD 3550–1						0.0515	0.1335	0.0238		0.3339	0.1812	0.0054	0.1758
MSD 3550–2						0.0012	0.0847	0.0257	0.0129	0.3914	0.3305	0.0097	0.3208
MSD2018817–0–5	1.1899	0.3217	0.0553	0.4253	0.0718	0.1037	0.0737	0.0793	0.0118	0.3347	0.4543	0.2717	0.1826
MSD2018817–5–10	0.7082	0.2309	0.0274	0.3609	0.0436	0.0757	0.1597	0.1063	0.0114	0.2308	0.4296	0.2071	0.2225
MSD2018817–10–20	0.6857	0.1222	0.0313	0.5675	0.0511	0.0943	0.3405	0.1105	0.0194	0.2122	0.4280	0.1259	0.3021
MSD2018817–20–30	0.6542	0.1915	0.0176	0.4772	0.0511	0.0804	0.2615	0.0957	0.0118	0.1843	0.3440	0.1742	0.1698
MSD2018817–30–40	0.7618	0.1743	0.0316	0.6501	0.0602	0.0708	0.4613	0.1064	0.0114	0.2249	0.3587	0.1630	0.1957
MSD2018817–40–50	0.6902	0.1698	0.0124	0.3605	0.0392	0.0556	0.1689	0.0810		0.1392	0.3267	0.1503	0.1764
MSD2018817–50–60	0.4446	0.1438	0.0408	0.3787	0.0310	0.0350	0.1983	0.0634	0.0168	0.1739	0.2761	0.1313	0.1448
MSD2018817–60–70	0.3979	0.0473	0.0140	0.2958	0.0236	0.0403	0.1584	0.0537	0.0151	0.2017	0.2606	0.0535	0.2071
MSD2018817–70–80	0.7588	0.0298	0.0199	0.4341	0.0314	0.0559	0.2374	0.0689		0.2665	0.3609	0.0388	0.3221
MSD2018817–80–90	0.7282	0.1637	0.0115	0.3353	0.0299	0.0494	0.1321	0.0618		0.2038	0.3801	0.1413	0.2388
MSD2018817–90–100	1.0485	0.0619	0.0183	0.2870	0.0259	0.0408	0.1206	0.0628		0.2052	0.2766	0.0623	0.2143
MSD2018817–100–110	1.2535	0.1492	0.0246	0.3539	0.0491	0.0449	0.1735	0.0835	0.0168	0.2347	0.4225	0.1400	0.2825
MSD MR 6	0.8555		0.0585	4.0219	0.2073	0.0623	2.1162	0.2232		0.4839	0.3952	0.0504	0.3448
MSD MR 7	1.7039		0.1414	8.2883	0.3353	0.0692	3.2399	0.1619	0.0206	0.2871	0.2938	0.0428	0.2510
MSD MR8	0.9301		0.0643	4.0838	0.2406	0.0535	1.1270	0.0746	0.0530	0.2521	0.1239	0.0330	0.0909

## 2 Experimental design, materials, and methods

Surface snow and snowpit samples were collected from Muz Taw glacier in July 15 and August 17, respectively. The melt snow samples were filtered through a 0.45 μm (pore size) PTFE filter for DOC and TDN analysis by using a total organic carbon analyzer in the laboratory [1], [2], [3] (Yan et al., 2016; Zhang et al., 2019). The detection limits for DOC and TDN were 4 and 5 μg *L*^−1^, respectively. The major ions in snow (including Na^+^, *K*^+^, NH_4_^+^, Ca^2+^, Mg^2+^, Cl^−^, SO_4_^2−^, NO_3_^−^, and NO_2_^−^) were analyzed in the laboratory via ion chromatography using a 200 μL sample loop [4] (Zhang et al., 2016) The DIN concentrations were calculated as the sum of the nitrogen concentrations from NH_4_^+^, NO_3_^−^, and NO_2_^−^ (Eq. (1)) [5](Wadham et al., 2016):

(1) $$\text{DIN}=\left[{\text{NO}}_{3}^{-}\right]+\left[{\text{NO}}_{2}^{-}\right]+\left[{\text{NH}}_{4}^{+}\right]$$

In this study, the DON concentrations were subsequently calculated by subtracting the corresponding DIN concentrations from TDN (Eq. (2)).

(2) DON=TDN−DIN

**Fig. 3:**
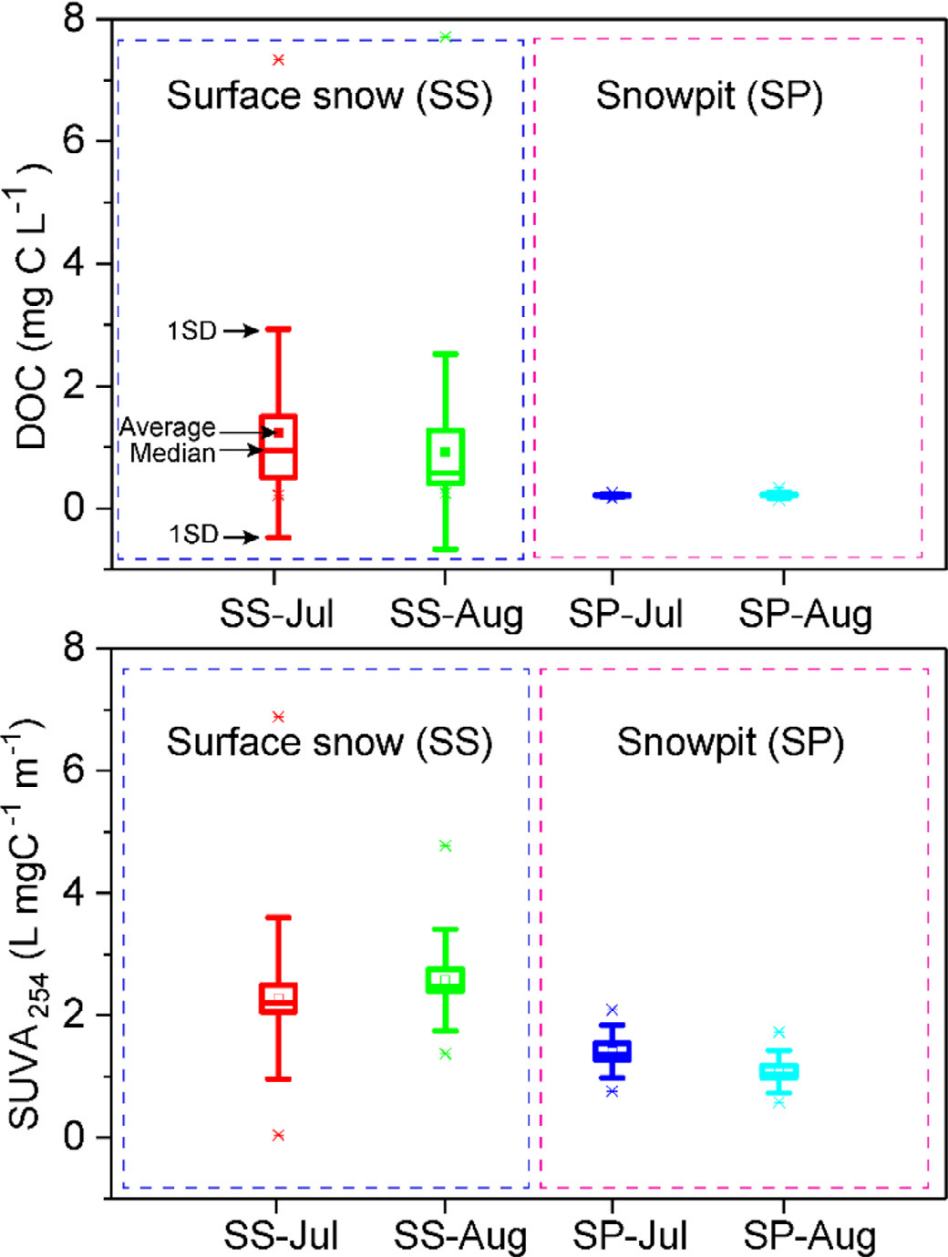
Statistic data of DOC concentrations and SUVA254 of DOC in snow from Muz Taw glacier.

SUVA_254_ of DOC was the division between the ultraviolet-visible absorption at 254 nm and the DOC concentration in snow (Fig. 3). The fraction (f) of RF by DOC relative to that by BC in snow was estimated as in Eq. (3) [6,7](Bosch et al., 2014; Kirillova et al., 2014):

(3) $$f=\frac{\int_{300}^{2500}I_{0}\left(\lambda \right)\times \left\{1-e^{-\left(MAC_{DOC365}\;{\left(\frac{365}{\lambda }\right)}^{AAE_{DOC}}\times \left[C_{DOC}\right]\times h_{ABL}\right)}\right\}d\lambda }{\int_{300}^{2500}I_{0}\left(\lambda \right)\times \left\{1-e^{-\left(MAC_{BC550}\;{\left(\frac{550}{\lambda }\right)}^{AAE_{BC}}\times \left[C_{BC}\right]\times h_{ABL}\right)}\right\}d\lambda }$$

Mass cross section of DOC (MAC_DOC365_) was calculated by the Eq. (4) [6,7](Bosch et al., 2014; Kirillova et al., 2014).

(4) $$\text{MA}C_{DOC365}=\frac{-ln\left|\frac{I}{I_{0}}\right|}{\left[C\right]\times L}=\frac{A}{\left[C\right]\times L}\times \text{ln}\left(10\right)$$

The obtained data were analysed using descriptive statistics such as average values of the detected chemicals (i.e., Fig. 3).

## Declaration of Competing Interests

The authors declare that they have no known competing financial interests or personal relationships which have, or could be perceived to have, influenced the work reported in this article.
